# Relatedness-based mate choice and female philopatry: inbreeding trends of wolf packs in a human-dominated landscape

**DOI:** 10.1038/s41437-024-00676-3

**Published:** 2024-03-12

**Authors:** Carolina Pacheco, Helena Rio-Maior, Mónia Nakamura, Francisco Álvares, Raquel Godinho

**Affiliations:** 1 CIBIO, Centro de Investigação em Biodiversidade e Recursos Genéticos, InBIO Laboratório Associado, Campus de Vairão, Universidade do Porto, Vairão, Portugal; 2 Department of Biology, Faculty of Sciences, University of Porto, Porto, Portugal; 3 BIOPOLIS Program in Genomics, Biodiversity and Land Planning, CIBIO, Campus de Vairão, Vairão, Portugal

**Keywords:** Inbreeding, Ecological genetics, Conservation biology

## Abstract

Inbreeding can reduce offspring fitness and has substantial implications for the genetic diversity and long-term viability of populations. In social cooperative canids, inbreeding is conditioned by the geographic proximity between opposite-sex kin outside natal groups and the presence of related individuals in neighbouring groups. Consequently, challenges in moving into other regions where the species is present can also affect inbreeding rates. These can be particularly problematic in areas of high human density, where movement can be restricted, even for highly vagile species. In this study, we investigate the socio-ecological dynamics of Iberian wolf packs in the human-dominated landscape of Alto Minho, in northwest Portugal, where wolves exhibit a high prevalence of short-distance dispersal and limited gene flow with neighbouring regions. We hypothesise that mating occurs regardless of relatedness, resulting in recurrent inbreeding due to high kin encounter rates. Using data from a 10-year non-invasive genetic monitoring programme and a combination of relatedness estimates and genealogical reconstructions, we describe genetic diversity, mate choice, and dispersal strategies among Alto Minho packs. In contrast with expectations, our findings reveal relatedness-based mate choice, low kin encounter rates, and a reduced number of inbreeding events. We observed a high prevalence of philopatry, particularly among female breeders, with the most common breeding strategy involving the pairing of a philopatric female with an unrelated immigrant male. Overall, wolves were not inbred, and temporal changes in genetic diversity were not significant. Our findings are discussed, considering the demographic trend of wolves in Alto Minho and its human-dominated landscape.

## Introduction

Mating between relatives (i.e., inbreeding) can reduce the fitness of offspring by unmasking deleterious alleles, a process known as inbreeding depression (Charlesworth and Willis 2009; Keller and Waller 2002). In many cooperatively breeding species, individuals live in family groups with established territories and often delay natal dispersal, resulting in the geographical clustering of opposite-sex kin beyond reproductive maturity (Hatchwell 2009; Lukas and Clutton-Brock 2012). Therefore, behavioural strategies, including reproductive restraint, extrapair reproduction, and sex-biased dispersal, can influence the rates and persistence of inbreeding in populations (Clutton-Brock and Lukas 2012; Leedale et al. 2020; Sparkman et al. 2012; see Nichols 2017 for a discussion on inbreeding tolerance). The latter strategy related to dispersal relies on the premise that different distances or rates of dispersal between sexes promote the geographical separation of females and males born in the same area (Pusey and Packer 1987). Notably, in canids, inbreeding within the natal group is apparently avoided (Geffen et al. 2011), whereas mating outside natal groups seems to occur randomly with respect to genetic, suggesting that, outside natal groups, geographical proximity of close kin is a preponderant driver of inbreeding (Ausband 2022a; Geffen et al. 2011). Therefore, inbreeding is of substantial conservation concern in small, isolated populations with high rates of sedentism or a predominance of short-distance dispersal, which leads to high local densities of relatives (e.g., Cockerill et al. 2022; Viluma et al. 2022). However, even in large contiguous populations, restrictions on movement can create localised areas with high densities of close relatives. Reduced movement behaviour is particularly prevalent in human-dominated landscapes, where habitat fragmentation can disrupt natural dispersal patterns (Morales-González et al. 2022; Rio-Maior et al. 2019; Tucker et al. 2018).

**Fig. 1:**
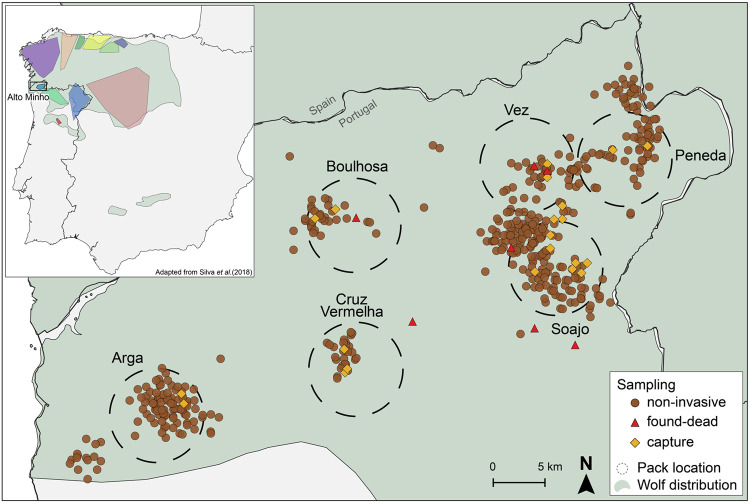
The geographical location of packs and samples used in this study. The inset depicts the location of Alto Minho within the wolf range in the Iberian Peninsula (green-shaded area), and the location of the genetically differentiated groups identified in the work of Silva et al. (2018). Pack territories are represented by dashed circles labelled with the respective pack name. Brown dots represent non-invasive samples (*N* = 470), red triangles represent individuals found dead (*N* = 6), and yellow rhombus represent the capture site of individuals captured for GPS-collaring purposes (*N* = 21).

Wolves are cooperative breeding canids that live in family groups (packs), typically consisting of a monogamous breeding pair and its offspring (Mech and Boitani 2003). Packs may also hold individuals unrelated to the breeding pair (i.e., immigrants), though their prevalence and sex ratio lack ample evidence (Jȩdrzejewski et al. 2005; Lehman et al. 1991; Rutledge et al. 2010). Juveniles and adults commonly disperse from their natal packs, with no consistent male-female differences (Morales-González et al. 2022). Wolf breeding pairs are generally unrelated (Caniglia et al. 2014; vonHoldt et al. 2008), yet inbreeding depression has been occasionally observed in small, isolated populations (Gómez-Sánchez et al. 2018; Hedrick et al. 2014; Liberg et al. 2005). Landscape resistance, due to environmental or anthropogenic factors, can limit gene flow in wolves, despite their common long-distance dispersal (Morales-González et al. 2022). The Iberian wolf (*Canis lupus signatus*) population inhabits a human-dominated landscape in the Northwest Iberian Peninsula, characterised by infrastructures, agriculture, and livestock production (Revelles et al. 2018). Compared to wolves in less altered habitats, Iberian wolves occur in a landscape with limited functional connectivity (Rio-Maior et al. 2019) and exhibit shorter average dispersal distances (Blanco and Cortés 2007; Nakamura et al. 2021). Based on genetic and spatial behaviour data, Silva et al. (2018) revealed that Iberian wolves exhibit spatially heterogeneous gene flow, resulting in genetically differentiated groups (inset Fig. 1) and a high prevalence of short-distance dispersal, mainly confined to the genetic group of origin. This population dynamics may increase the likelihood of inbreeding by promoting encounters between opposite-sex relatives outside their natal groups, particularly in geographically smaller genetic groups with limited gene flow with neighbouring regions of wolf presence (Silva et al. 2018; see inset Fig. 1). In this regard, the Iberian wolf provides an interesting study system to understand the effect of low population connectivity on local mate selection patterns and inbreeding levels.

In this study, we assess the influence of socio-ecological dynamics on the genetic diversity and inbreeding levels of Iberian wolf packs within a genetically differentiated group with a high prevalence of short-distance dispersal. To achieve this, we used time-series genetic data recovered from a 10-year monitoring programme and employed relatedness estimates and pedigree reconstruction methods to answer the following questions: Is inbreeding recurrent? Does relatedness influence mate choice? How likely is it for a disperser to find a close relative? What are the individual strategies for breeding-pair formation? How often are unrelated individuals adopted into packs? We hypothesise that inbreeding is common among these wolves and predict that this trend would be linked to two factors: a higher kin encounter rate compared to populations with no restriction of movement and random mating with respect to genetic relatedness, as described for the species.

## Material and methods

### Study area

Our study focuses on the Alto Minho region in northwest Portugal (Fig. 1). Wolves inhabiting this region are genetically differentiated from the remaining Iberian wolf population, and their gene flow rates with neighbouring regions are not significantly different from zero (Silva et al. 2018). Alto Minho is a mountainous region characterised by high human population density (110.3 inhabitants/km^2^; PORDATA 2020) and a heterogeneous landscape dominated by production forest (68%), cultivated land and pastures (28%; CORINE Land Cover 2012). As for the rest of Iberian Peninsula, wolves in this region are protected through National and European legislation (e.g., Bern Convention Annex II, EU Habitats Directive Annex IV). The wolf diet in the region is based primarily on livestock, mostly free-ranging horses and cattle, which often leads to conflicts and high levels of illegal persecution (Pimenta et al. 2018). These conflicts and the increasing human influence on the landscape result in a high risk of human-caused mortality for Alto Minho wolves (Rio-Maior et al. 2018).

### Sampling and genotyping

Alto Minho wolves have been the target of a >20-year monitoring programme that, since 2008, has included the molecular identification of individuals. In this study, we analysed the genetic profiles of 150 wolves collected between 2008 and 2017 in the framework of this monitoring program. We selected these genetic profiles based on data quality criteria, focusing on samples with less than 25% missing data. This data was generated from 470 non-invasive genetic samples (gNIS; Nakamura et al. 2017; Nakamura et al. 2021), 21 individuals captured for GPS-collaring purposes (Rio-Maior et al. 2018, 2019), and six individuals found dead (Fig. 1). Six packs were identified in the region (Nakamura et al. 2021), with stable territories throughout our sampling period, as defined by GPS telemetry from the home ranges of resident wolves (Nakamura et al. 2021; Rio-Maior et al. 2019). Sampling and individual genotyping used in this work were performed in the scope of the wolf monitoring program and are detailed in Nakamura et al. (2017, 2021). Briefly, gNIS comprising mostly scats, were collected along transects and homesites, encompassing the territory of all six packs. The transects were defined along unpaved roads and paths throughout the landscape and were conducted monthly, seasonally or during autumn-summer, with an average sampling effort of 95.2 km per pack per year (Nakamura et al. 2017, 2021). Homesites were estimated by combining information from GPS telemetry with visual or acoustic detection of pups from late July to September (Rio-Maior et al. 2018). When detected, the homesites were sampled by a 2-person crew in one session, during which all detected samples were collected. One homesite per pack was surveyed in October, right after pup-rearing season when pups are more mobile, and packs are expected to be less sensitive to disturbance at breeding sites (e.g., Stenglein et al. 2011). DNA extracts were obtained using the GuSCN/silica procedure (Boom et al. 1990) for scats or the DNeasy Blood & Tissue Kit (Qiagen) for blood and muscle. All samples were sequenced for a mitochondrial DNA (mtDNA) control region fragment for species identification and genotyped for 19 microsatellites, selected among the most informative for individual identification in Iberian wolves (Godinho et al. 2011, 2015; Nakamura et al. 2017, 2021). The average allelic drop-out rate was 10.6%, whereas the false allele rate averaged 0.7% across loci (Nakamura et al. 2021). To dismiss potential wolf-dog hybridisation cases (Godinho et al. 2011), a Bayesian clustering analysis was implemented in Structure 2.3.4 (Pritchard et al. 2000) using the reference database and procedures described in Pacheco et al. (2017). The software Arlequin 3.5 (Excoffier and Lischer 2010) was used to estimate departures from Hardy–Weinberg equilibrium following Guo and Thompson (1992) and to evaluate the significance of association between genotypes at pairs of loci (linkage disequilibrium, LD). Statistical significance was adjusted using sequential Bonferroni corrections.

### Pedigree reconstruction

To reconstruct the genealogy of Alto Minho wolves, we applied the full-likelihood method implemented in Colony v2.0.6.4 (Jones and Wang 2010), allowing for inbreeding and assuming locus-specific allelic dropout rates. Wolves only sampled non-invasively, for which age was unknown, were considered both as potential parents and offspring in the analysis. In contrast, for GPS-collared and found dead wolves, we considered them as potential parents or offspring depending on the estimated age at capture or death. To overcome possible bias in genealogy inference due to fluctuations in allele frequencies over generations (Wang 2011b), we sub-sampled our data using a sliding window approach (window size = 3 years; step size = 1 year), resulting in eight datasets, each with 50–90 individuals. Each dataset was then analysed in Colony using the respective allele frequencies. These analyses were performed in chronological order, including *known* paternal and maternal siblings established in the previous steps, thus increasing accuracy for the inferred sibling groups. Only full-sibling families with probability of inclusion equal to or higher than 0.85 were considered. As a complementary approach to ensuring that all possible relationships were identified, we also performed a Colony analysis, including all individuals as potential offspring and all males and females as potential fathers and mothers, respectively. Concordant relationships across the different analyses were included in a consensus genealogy, further corroborated by matching mother and offspring mtDNA haplotypes and information for sex, age, and pack assignment available from the GPS-tracked individuals. Finally, we also calculated the effective population size (N_e_) of Alto Minho wolves in Colony using the whole dataset and the sibship assignment method, which estimates the current N_e_ in a subpopulation with immigration (Wang 2009).

### Relatedness and genetic diversity

To determine the best performing relatedness estimator for our dataset, we first simulated 3000 dyads (i.e., pairs of individuals) representing six different relationships: parent-offspring, full-siblings, half-siblings, first cousins, second cousins, and unrelated individuals (500 dyads each). To run these simulations, we used the software Coancestry 1.0 (Wang 2011a) and the observed allele frequencies for the whole Alto Minho dataset. Second, we estimated the genetic relatedness of simulated dyads using the five moment and two maximum-likelihood estimators available in Coancestry 1.0, as well as the maximum-likelihood estimator implemented in ML-Relate (Kalinowski et al. 2006). To find the most accurate estimator, we used Pearson correlation coefficients to compare the estimated genetic relatedness values with the real values. After conducting this comparison, we found that the triadic maximum-likelihood estimator (TrioML; Wang 2007) performed the best (Pearson’s r = 0.801; see Supplementary Table S2). We then used TrioML, implemented in Coancestry 1.0, to estimate pairwise genetic relatedness (*r*) between all pairs of individuals and individual inbreeding coefficients (*F*) for all Alto Minho wolves.

To establish a threshold for classifying dyads as related or unrelated based on the estimated pairwise relatedness values, we followed the method described by Blouin et al. (1996) and Lucchini et al. (2002). This threshold was defined as the midpoint value between the averages of the distributions of the TrioML relatedness values for the simulated unrelated and first-order relative dyads (parent-offspring plus full-siblings). To test whether Alto Minho wolves avoid inbreeding, we compared relatedness between real and randomly generated mating partners using the Monte Carlo simulation approach implemented in Storm (Frasier 2008). Simulations were performed over 1000 iterations, using all sampled females and males as potential mothers and fathers, respectively. In each iteration, the number of simulated breeding pairs was the same as that identified with the reconstructed genealogy. We then calculated the average relatedness of the simulated breeding pairs in each iteration and compared the resulting distribution with the average relatedness of real breeding pairs. Statistical significance was determined considering an alpha of 0.05.

We estimated the genetic diversity of Alto Minho wolves based on observed (Ho) and expected (He) heterozygosity using the diveRsity package in R (Keenan et al. 2013; R Core Team 2020). These statistics are presented for the entire dataset on an annual basis (i.e., based on all detected animals per calendar year), and per social group (Parreira and Chikhi 2015; Parreira et al. 2020), defined here as all individuals assigned to the pack during the tenure of each breeding pair. To assess significant differences between consecutive years or social groups, we conducted Kruskal-Wallis tests.

### Inference of social organisation and dispersal

Individuals were considered pack residents according to three criteria used in a recent study targeting the same packs (Nakamura et al. 2021): i) were confirmed as pack members by GPS telemetry; or were detected through gNIS ii) at least once in the homesite, or iii) at least twice a year inside the pack territory. Exceptions to these criteria were allowed only when the individual’s ancestry was determined through their genealogy. Individuals that were sampled for at least two years and had a confirmed natal pack based on the genealogy reconstruction were classified as philopatric if only sampled in the natal pack or as immigrants if identified as residents in a different pack.

To estimate the overall probability of kin encounters across our study period, we calculated the proportion of full or half-sibling male-female pairs (based on the genealogy) relative to all possible pairs among all sampled individuals, following the method described by Geffen et al. (2011). Additionally, we estimated the same probability considering only individuals identified as dispersers, i.e., the proportion of possible full or half-sibling male-female pairs among dispersers relative to the total number of possible pairs among dispersers.

Based on the genealogy and the intensive annual sampling, we assumed that offspring were born in the first year they were detected when sampled after the breeding season (i.e., after May; Rio-Maior et al. 2018) or otherwise were born in the previous year. This information was then used to estimate the approximate bond duration (in years) for each breeding pair. Additionally, we looked for evidence of polygamy or multiple breeding pairs (i.e., more than one female in the pack giving birth in the same year). Finally, for wolves identified as dispersers, the minimum age at dispersal was assumed to be the time interval between estimated birth and the date of the first recapture in the new pack.

## Results

### Loci diversity and individual identification

All microsatellite loci were polymorphic, with an average of five alleles per locus, ranging from two to seven (Table S1). Deviations from Hardy–Weinberg expectations were observed in three loci (*p* < 0.001) and several pairwise comparisons deviated significantly from linkage equilibrium (Supplementary Table S1). However, no loci were removed from the analysis because previous LD evaluations for the same markers across the entire Iberian wolf population did not result in deviations from expectations (Godinho et al. 2015). Initial screening for wolf-dog hybridisation revealed no evidence of admixture in our sampling (Supplementary Fig. S1). The 150 wolves analysed in this study presented a sex ratio of 1:1 (74 M:75 F; one unknown). On average, each wolf was sampled 3.21 ± 2.76 (s.d.) times, with some individuals sampled up to 16 times. When considering only non-invasive samples, on average, wolves were sampled 2.06 ± 2.48 (s.d.) times in transects and 1.09 ± 1.62 times at homesites. Notably, 33% of the individuals were exclusively sampled at homesites, while 49% were exclusively sampled in transects. Considering the 101 individuals sampled more than once, 76% (*N* = 77) were continuously sampled within the same pack territory, whereas 23% (*N* = 23) were sampled in two packs, and a single individual in three pack territories. The average sampling period per individual was 1.7 ± 1.2 (s.d.) years, and 34% (*N* = 51) of the individuals were sampled for two or more years, up to seven years (Supplementary Fig. S2). Temporal trends of heterozygosity and inbreeding coefficient for Alto Minho wolves revealed a stable pattern across years (2008 to 2017), with overall averages of H_o_ = 0.60 ± 0.16 (s.d.) and H_e_ = 0.58 ± 0.14 (s.d.) and no significant differences between consecutive years (Supplementary Fig. S3). Two mtDNA haplotypes known for Iberian wolves, namely W6 and W7 (Valière et al. 2003), were observed with a frequency of 89% (*N* = 136) and 11% (*N* = 16), respectively.

### Pack composition and effective population size

**Fig. 2:**
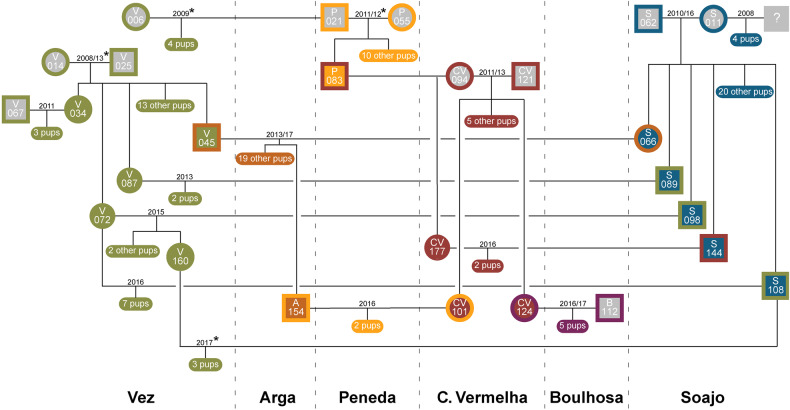
Genealogy of the Alto Minho wolves. Horizontal lines connect breeding pairs, with the years of the pair’s bond shown above the line. Vertical lines indicate individuals’ ancestry. The number of non-breeding offspring is indicated for each breeding pair. Packs are represented in different colours (Vez: green; Arga: light brown; Peneda: yellow; Cruz Vermelha: red; Boulhosa: violet; Soajo: blue). Individuals with unknown ancestry are depicted in grey. Each icon represents a breeding individual. Squares denote males, and circles denote females. The icon’s fill colour indicates the natal pack, whereas the outline colour represents the breeding pack. ID letters correspond to the natal pack name or where the individual has always been sampled. Asterisks denote the inbred pair bonds.

The final genealogy included 127 individuals (~85% of all sampled individuals; Fig. 2). Six of the 23 individuals not included in the genealogy were classified as pack residents. The remaining 17 individuals were only detected once during the first three years of our sampling period outside or in overlapping pack territories. We considered them to be individuals from past family lineages since relatedness estimates indicate these are related to each other but also to individuals included in the genealogy, mainly to earlier breeders. We detected 16 social monogamous breeding pairs, comprising 27 wolves (14 males and 13 females). In one of the 16 breeding pairs, only the female was sampled. The average pair bond duration was 2.3 ± 1.8 (s.d.) years, ranging from 1 to 7 years (Supplementary Fig. S4). Packs included the breeding pair and up to 12 related members, including offspring of the year, yearlings (offspring of previous years) and other non-first-degree relatives, plus up to 3 unrelated individuals (Supplementary Fig. S5). The cumulative number of offspring per breeding pair ranged from 1 to 25, with an annual average of 3.7 ± 1.8 (s.d.). Seven unrelated individuals (5 M:2 F) were detected as residents in four out of the six packs (~70%) for at least one year, up to four years (Supplementary Fig. S5). Across all packs, the average ratio of unrelated pack members was 20% (Supplementary Fig. S5). The average annual pack size, including unrelated individuals, was 8.9 ± 3.2 (s.d), and the current effective population size was estimated to be 15 (_95%_CI: 9, 34) assuming random mating and 16 (_95%_CI: 8, 30) assuming non-random mating.

### Relatedness and inbreeding

Simulated pairwise relatedness for unrelated ($\bar{x}$ = 0.006 ± 0.214) and for first-order relatives (parent-offspring and full-siblings; $\bar{x}$ = 0.487 ± 0.164) resulted in a partially overlapping distribution (Supplementary Fig. S6). We define the threshold for classifying a dyad as related at 0.240, finding that 4% of the unrelated and 8% of the first-degree relatives would be misclassified. The average pairwise relatedness among Alto Minho wolves was *r* = 0.108 ± 0.163 (s.d.; Supplementary Fig. S7). Most breeding pairs for which we had both the female and male genotypes were unrelated (*N* = 12; 80%; Table 1). Two breeding pairs were distant relatives (Vez pack; Table 1), whereas a single breeding pair was formed by first-order relatives (*r* = 0.5; P021 × P055, Peneda pack; Table 1, Fig. 2). Additionally, based on the genealogy, we detected one breeding pair between second-order relatives (a niece and an uncle; V160 × S108 in Vez pack, Fig. 2), which presented a relatedness value of zero. Despite the observed cases of inbreeding, we found evidence for relatedness-based mate choice, as the relatedness of real mating pairs was significantly lower (*p* < 0.013; Supplementary Fig. S8) than that of randomly generated mating pairs. Alto Minho wolves presented an average inbreeding coefficient of *F* = 0.059 ± 0.107 (s.d.), whereas breeding pairs had an average of *F* = 0.03 ± 0.05 (s.d.; Table 1).

**Table 1:** Genetic information of the 15 breeding pairs with known female and male genotypes.

Territory	Breeding Pair		Relatedness (TrioML)		Inbreeding coefficient		Obs Heterozygosity		Bond duration (years)	1^st^ year of reproduction	# Offspring
	Female	Male	*r*	CI	Female	Male	Female	Male			
Vez	V006	P021	0.18	(0.02, 0.71)	0.02	0.01	0.65	0.57	1	2009	4
Vez	V014	V025	0.17	(0, 0.54)	0.10	0.00	0.42	0.63	6	2008	17
Vez	V034	V067	0.03	(0, 0.50)	0.02	0.01	0.53	0.56	1	2011	3
Vez	V087	S089	0.00	(0, 0.24)	0.00	0.08	0.58	0.60	1	2013	4
Vez	V072	S098	0.00	(0, 0.04)	0.20	0.00	0.42	0.79	1	2015	3
Vez	V072	S108	0.00	(0, 0.09)	0.20	0.00	0.42	0.68	1	2016	7
Vez	V160	S108	0.00	(0, 0.23)	0.00	0.00	0.79	0.68	1	2017	3
Arga	S066	V045	0.00	(0, 0.08)	0.00	0.01	0.74	0.58	5	2013	20
Peneda	P055	P021	0.50	(0.23, 0.64)	0.00	0.01	0.73	0.57	2	2011	11
Peneda	CV101	A154	0.00	(0, 0.12)	0.00	0.00	0.74	0.63	1	2016	2
Cruz vermelha	CV094	P083	0.00	(0, 0.15)	0.07	0.03	0.74	0.63	1	u	1
Cruz vermelha	CV094	CV121	0.00	(0, 0.10)	0.07	0.17	0.74	0.42	3	2011	7
Cruz vermelha	CV177	S144	0.00	(0, 0.13)	0.00	0.00	0.95	0.74	1	2016	2
Boulhosa	CV124	B112	0.00	(0, 0.09)	0.01	0.00	0.63	0.58	2	2016	5
Soajo	S011	S062	0.00	(0, 0.16)	0.00	0.04	0.53	0.68	5	2010	25

Breeding pair relatedness (*r*) and confidence intervals obtained with the TrioML estimator are presented, along with the inbreeding coefficient and observed heterozygosity of each individual breeder. Pair bond duration (number of years), year of the first reproduction, and total number of offspring are also presented. *u* undefined.

### Genetic diversity of full sibling families

**Fig. 3:**
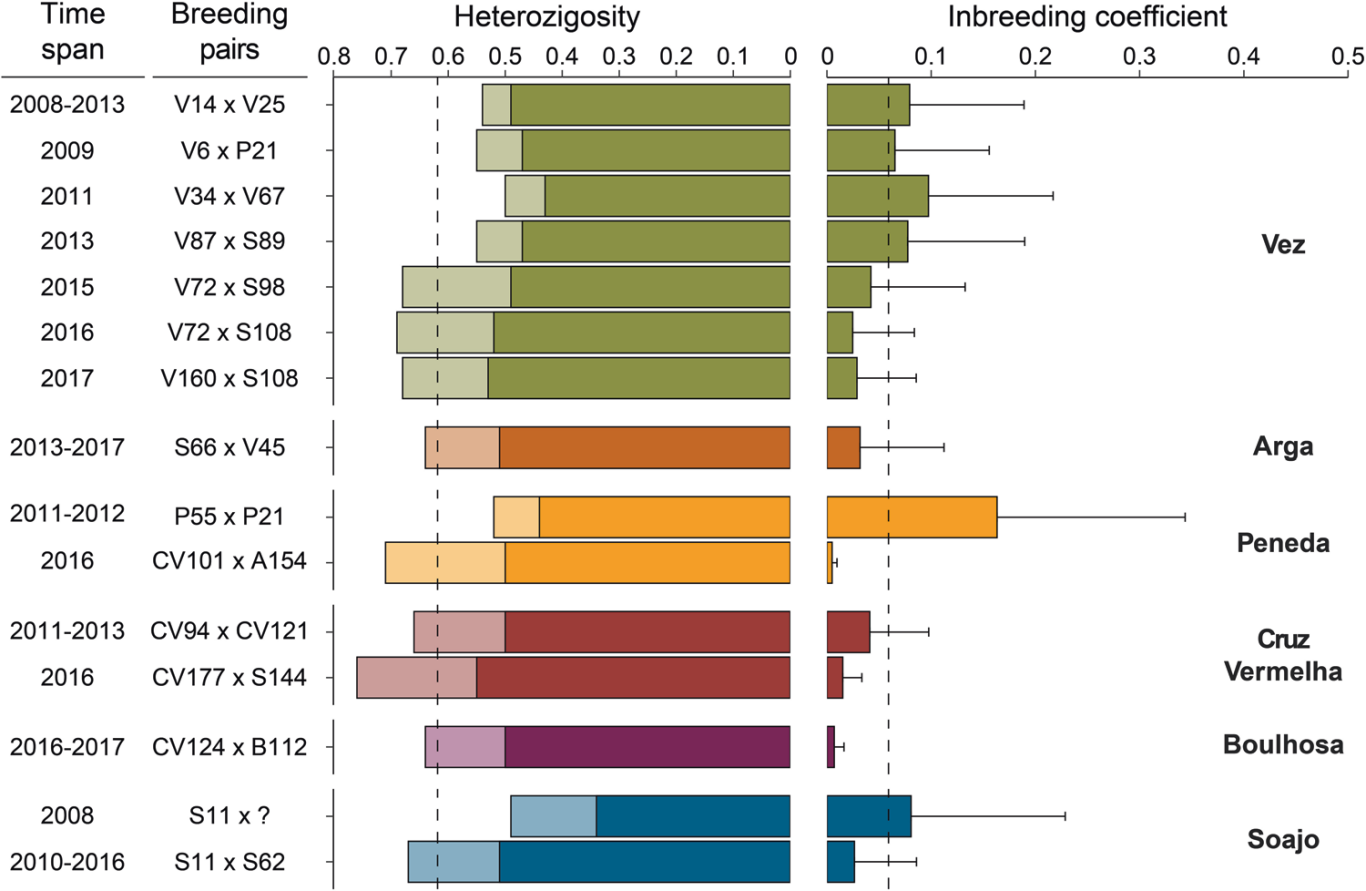
Distribution of heterozygosity and inbreeding coefficient estimates per social group. Observed (lighter shade) and expected (darker shade) heterozygosity are depicted on the left panel, and average individual inbreeding coefficients on the right panel. The progenitors’ IDs and the time span of each pair bond are identified along the y-axis. Each colour depicts a distinct pack (as in Fig. 2) whose name is identified on the right side of the plot. Dashed lines represent the overall average estimates of observed heterozygosity and inbreeding coefficient for Alto Minho wolves.

On average, social groups in Alto Minho showed low inbreeding coefficients (*F* = 0.036) and high observed heterozygosity (H_o_ = 0.62; H_e_ = 0.48; Fig. 3). However, six families revealed an opposite pattern, exhibiting reduced observed heterozygosity and increased inbreeding coefficient (Fig. 3). This was the case for the offspring of the first-order relative breeding pair in the Peneda pack, represented by two full-siblings with the highest estimated inbreeding value among Alto Minho wolves (*F* = 0.537 and 0.418). Three full sibling families in the Vez pack and one in the Soajo pack also presented low genetic diversity and high inbreeding. As a general pattern, offspring of breeding pairs established earlier in our sampling seem to present lower genetic diversity than those established later (Fig. 3).

### Inter-pack dispersal

**Fig. 4:**
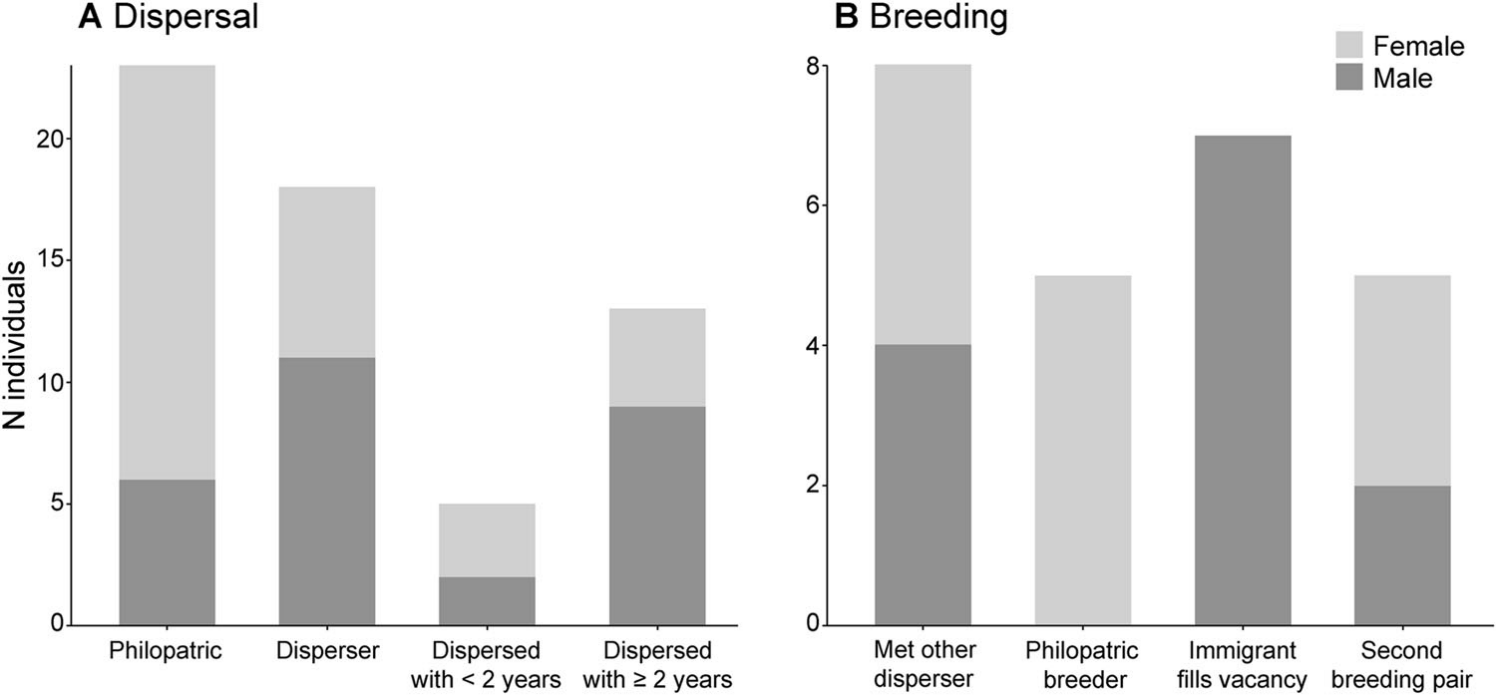
Dispersal and breeding strategies observed for female and male wolves in Alto Minho. Panel **A** depicts the frequency of the different individual dispersal strategies, and panel **B** depicts the frequency of the different strategies adopted to establish a new pair bond. Note that the same individual can be considered for multiple categories.

We identified 23 philopatric individuals with a significant female-biased trend (17 F:6 M; *χ*^2^ = 8.69, df = 1, *p*-value = 0.0016), of which 5 (22%; 5 F:0 M) became breeders. Additionally, we identified 18 dispersers (7 F:11 M, no significant sex-bias trend), of which eleven (61%; 4 F:7 M) became breeders in the new pack (Fig. 4A). Considering all individuals, the estimated kin encounter rate with a full or half sibling was 0.067 (_95%_CI: 0.061, 0.074) and 0.016 (_95%_CI: 0.013, 0.019), respectively. The kin encounter rate considering only dispersers was 0.247 (_95%_CI: 0.150, 0.343), which is significantly greater than considering all possible pairs (*χ*^2^ = 23.8, df = 1, *p*-value = 5.340 × 10^−7^). Still, we did not find any evidence of reproduction between these siblings, and only once opposite-sex dispersing siblings were detected simultaneously in the same pack. We identified both emigrants and immigrants in all packs, except for the Boulhosa pack, where only immigrants were observed. Based on the period each wolf was detected in the natal pack before dispersal, we categorised dispersers into two possible age classes: juveniles (i.e., 12–23 months; detected less than two years in the natal pack; *N* = 5; 3 F:2 M); and adults (i.e., ≥24 months; detected for at least two years in the natal pack *N* = 13; 4 F:9 M; Fig. 4A).

### Breeding-pair formation

Based on 13 breeding pairs with known ancestry that have been established after 2008 (Fig. 2), we identified four main strategies for forming new pair bonds among Alto Minho wolves: (1) joining of two dispersers (*N* = 4); (2) replacement of one breeder either by an offspring or an immigrant (i.e., unrelated dispersal from another pack; *N* = 4); (3) replacement of both breeders by an offspring and an immigrant (*N* = 2) and (4) formation of a second breeding pair inside an existing pack territory, involving an offspring and an immigrant (*N* = 3; Fig. 4B). Strategy (1) is represented by dispersers that occupied vacant territories, i.e., territories with no evidence of breeding packs during the previous years (according to Nakamura et al. (2021); e.g., S066 × V045 in Arga pack, Fig. 2). In contrast, the establishment of breeding pairs through strategies (2), (3) and (4) was consistently associated with the reproduction of a philopatric female with an unrelated male (Figs. 2 and 4B). Strategy (2) was represented by three cases of replacement of the breeding male by an immigrant (e.g., V072 × S108 in Vez pack, Fig. 2) and one case of replacement of the breeding female by her daughter (V160 x S108 in Vez pack, Fig. 2), who was not related to the breeding male. In the latter case, the replaced female remained in the pack. Regarding the replacement of the breeding males, we could only assess the fate of one individual, which was sampled in multiple packs in the following breeding season. Strategy (3) involved daughters of the previous breeding pairs (e.g., V072 × S098 in Vez pack, Fig. 2) that established a pair bond with an immigrant. Finally, in strategy (4), breeding pairs were composed of a daughter of the resident breeding pair and an immigrant (e.g., V034 × V067 in Vez pack, Fig. 2). This strategy was only observed in the Vez pack, with both litters detected in the same rendezvous (Supplementary Fig. S4; Rio-Maior et al. 2018).

## Discussion

In cooperative breeding species, particularly in canids, inbreeding is more prone to occur outside the natal group, especially when dispersers are likely to encounter opposite-sex kin (Geffen et al. 2011; Nichols 2017; Pike et al. 2021). Therefore, the prevalence of short-distance dispersal described for Iberian wolves from genetically differentiated groups, including those from Alto Minho (Silva et al. 2018), could potentiate inbreeding. In contrast to our initial prediction, kin encounter rates among Alto Minho wolves were relatively low, and inbreeding does not seem to be recurrent, as it was mostly observed during the first sampling years. Additionally, the general assumption that wolves mate randomly with respect to relatedness outside natal packs (Ausband 2022a; Geffen et al. 2011) was not confirmed in Alto Minho, where we found that relatedness among breeding pairs was significantly lower than expected under random mating conditions.

We observed inbreeding in 26% of the detected breeding pairs. This indicates that inbreeding in Alto Minho is more frequent than that described for populations in less disturbed landscapes, with or without harvesting, like the Italian Apennine (3%; Caniglia et al. 2014), the Idaho Rocky Mountains (6%; Ausband 2022a), Yellowstone (7%; vonHoldt et al. 2008), or southern Finland (15%; Granroth-Wilding et al. 2017). However, the detected inbreeding rate is similar to that described for the central Idaho population before harvest (25%; Stenglein et al. 2011). Moreover, Alto Minho’s inbreeding rate is comparable to that described for the expanding German population based on the full pedigree inbreeding coefficients (~30%; Jarausch et al. 2021) and considerably lower than those observed for the inbred Scandinavian wolf population (87%; Åkesson et al. 2016; Liberg et al. 2005). An interesting pattern that arises from the observed inbreeding events in Alto Minho is that most of the inbred pair bonds (75%) occurred during the first sampling years (2008–2011). The beginning of our sampling follows a period of steep population decline between 1996 and 2005 (growth rate = −8%) when the Alto Minho wolf population was reduced to two breeding packs (Vez and Soajo; Nakamura et al. 2021). In combination with the reduced dispersal to and from elsewhere in Iberia (Silva et al. 2018) and the limited functional connectivity between Alto Minho packs (Rio-Maior et al. 2019), mating opportunities with nonrelatives would likely be scarcer during this period. After 2005, there was a shift in the demographic trend, and the population started to grow in number of individuals and breeding packs (Nakamura et al. 2021). Such a shift likely triggered an increase in mating opportunities with nonrelatives. This ultimately contributed to the observed predominance of unrelated mating pairs over the most recent sampling years.

Interestingly, despite our expectations, we uncovered a low overall kin encounter rate (7%) among Alto Minho wolves. This estimate is comparable to those described in outbred populations (3 to 7%; Geffen et al. 2011) and lower than in inbred populations (Isle Royale; 20%; Geffen et al. 2011). In contrast, the potential kin encounter rate among dispersers was significantly higher, with one in every four potential pairs being half or full-siblings. However, only once opposite-sex dispersing siblings were detected together in the same pack, and no breeding events were detected among these individuals. Different dispersal timings and distances among opposite-sex dispersing siblings may help explain such a pattern (Nichols 2017). However, it is important to note that the movement behaviour of Alto Minho wolves is shaped by the active avoidance of human activities and infrastructures (Rio-Maior et al. 2019) and that these wolves experience a low annual survival rate (0.54; _95%_CI: 0.35, 0.83), mostly associated with road-killing and poaching (Barroso et al. 2016; Nakamura et al. 2021; Rio-Maior et al. 2018). Such evidence was used to suggest that wolves in Alto Minho face a high risk of mortality when dispersing (Nakamura et al. 2021; Rio-Maior et al. 2019), which may also help explain the reduced “effective” probability of encountering a sibling outside the natal pack here described.

Regarding the dispersal patterns among Alto Minho packs, we found a slightly higher proportion of philopatric individuals than dispersers. Also, we found evidence for delayed dispersal, as most of the dispersers we detected were likely older than the most common dispersal age of 11 to 24 months described for the species (Blanco and Cortés 2007; Mech and Boitani 2003; Morales-González et al. 2022), though a mean dispersal age of 3 years has been reported for wolves in the Rocky Mountains (Jimenez et al. 2017). In wolves, longer permanence in the natal pack has been associated with areas of high prey availability (Ballard et al. 1987). Additionally, it has been suggested to increase the opportunity to learn subtle components of hunting and foraging behaviours (Mech and Boitani 2003), which might be particularly beneficial in a human-dominated landscape (Rio-Maior et al. 2019). In fact, in the Rocky Mountains population, age at dispersal has been identified as a strong predictor of dispersal success (defined as a dispersing wolf that reproduces; Jimenez et al. 2017). Additionally, delayed, or reduced dispersal behaviour has been associated with higher human-related mortality risk in wolves (Adams et al. 2008; Sells et al. 2022) and other large carnivores (Elliot et al. 2014; Sparkman et al. 2011).

In *Canis* species, philopatric breeding has been associated with a high risk of inbreeding unless unrelated mating opportunities are frequent within the natal pack (Randall et al. 2007; Robinson et al. 2019; Sparkman et al. 2012). Whereas records of unrelated individuals being adopted into a pack are not uncommon for wolves (Mech and Boitani 2003), the proportion of packs with immigrants varies across populations, ranging from sporadic (Jȩdrzejewski et al. 2005; Stansbury et al. 2016) to as high as 80% (Rutledge et al. 2010). In Alto Minho, we detected unrelated immigrants in four out of six packs, comprising, on average, 20% of the total pack size. This result indicates that the presence of unrelated individuals in Alto Minho packs is relatively common, though the direct comparison of this proportion with other studies is precluded by the implementation of different methodologies. The proportion of immigrants within packs is likely influenced by the combined effect of social structure, population density, and persecution intensity, as suggested by Bassing et al. (2020). Still, higher proportions of immigrants are often associated with populations experiencing high human-related mortality (Adams et al. 2008; Ballard et al. 1987; Larivière et al. 2000), where unrelated individuals can comprise up to 46% of breeding packs (Grewal et al. 2004). In Alto Minho, an association between human-related persecution and the proportion of unrelated individuals in packs may not be excluded; however, further data on the mortality per pack would be needed to test it. Given wolf’s social structure and territoriality, the acceptance of unrelated individuals into the pack may suggest an increased intraspecific tolerance (Mech 1994; Mech 1993). This would be in line with the plausible rareness of intraspecific killing events among Alto Minho wolves (Barroso et al. 2016), in contrast to most wolf populations, particularly in North America (Mech 1970). Additionally, the presence of unrelated immigrants in packs offer philopatric individuals the chance to breed without inbreeding. Notably, in three out of the four inbreeding events observed in Alto Minho, there were no unrelated individuals in the pack. In the remaining case, a female unrelated to the breeding male was present. Altogether, these observations may be related to the prevalence of the short-distance dispersal observed for Iberian wolves.

Wolves can display various strategies for establishing breeding pairs (Mech and Boitani 2003; vonHoldt et al. 2008). One of the most common involves the pairing of two dispersers in a new or vacant territory (Fritts and Mech 1981; Hayes and Harestad 2000). In Alto Minho, we found this to be one of the most common strategies, but equally frequent as the replacement of one breeder in an established pack. When strategies involved replacing one or both breeders or a second breeding pair in the pack, it most often resulted from the pairing of a philopatric female with an unrelated male. Female wolves seem to have a higher probability of becoming breeders in the natal pack, as also described across many other populations, including in Yellowstone National Park, Italy, Germany, and Poland (Ausband et al. 2017; Caniglia et al. 2014; Jȩdrzejewski et al. 2005; Mech and Boitani 2003; vonHoldt et al. 2008). This trend is likely associated with sex-specific fitness benefits of philopatry, such as maternal nepotism (Lynch et al. 2019; vonHoldt et al. 2008), making females the sex that would benefit most from remaining in the natal pack and breeding with an immigrant. Notably, in Alto Minho, we found evidence of female-biased philopatric behaviour and breeding; in contrast, 61% of dispersers, all males, became breeders in the new pack. Whereas this trend is not uncommon in wolf populations, it is mostly documented in saturated habitats, whether due to high wolf population densities or human-dominated landscapes (Ausband 2022b; Caniglia et al. 2014; Jarausch et al. 2021; Jȩdrzejewski et al. 2005; vonHoldt et al. 2008), the latter representing the case in Alto Minho. Additionally, in the study area, this pattern may reflect the fitness benefits of sex-specific philopatry, possibly acting as a mechanism to deal with the perceived risks and limited connectivity of a human-dominated landscape (Rio-Maior et al. 2019) while avoiding inbreeding. Furthermore, the variety and dynamics of breeding strategies observed in Alto Minho may have contributed to the stability of genetic diversity levels through time, particularly when associated with the occurrence of multiple breeding pairs in a group, as also suggested by Ausband (2018) for North American wolf populations. In Alto Minho, such can be exemplified by the genetic diversity increase following the replacement of Soajo’s breeding male or the occurrence of multiple breeding pairs in Vez. Overall genetic diversity of Alto Minho wolves seems to be slightly higher than what has been previously described for the region by Silva et al. (2018; H_o_ = 0.53; H_e_ = 0.52), but comparable to the temporal fluctuations’ ranges described for the expanding German wolf population (Jarausch et al. 2021).

## Conclusion

This study documents the social ecology dynamics of wolves with prevalence of short-distance dispersal inhabiting a human-dominated landscape. Despite the perceived challenges of this landscape and our expectations, we found that inbreeding was not recurrent and overall genetic diversity is high. Accordingly, we also found that Alto Minho wolves experience low kin encounter rates and show a significant bias towards unrelated breeding patterns. Wolves in Alto Minho exhibit several strategies to establish a breeding pair, the majority of which involving a philopatric female mating with an immigrant male. It is possible that the latter is associated with sex-specific fitness benefits. Overall, our findings contribute to the increasing understanding of the intricate patterns of wolf pack dynamics and how such mechanisms contribute to their persistence in human-dominated landscapes.

### Supplementary information

Supplemental information

## Data Availability

The wolf genotypes used in this study and information on the social status of each individual across sampling years can be accessed through The Open Science Framework using the following link: https://osf.io/p89rn/?view_only=5f7c5bf6cb574f3b8fcade0ad15c0524.
