# Supplementary material for: Relatedness-based mate choice and female philopatry: inbreeding trends of wolf packs in a human-dominated landscape
Source: Heredity (Edinb). 2024 Mar 12;132(4):211–20. doi: 10.1038/s41437-024-00676-3 (PMC10997798; doi:10.1038/s41437-024-00676-3)
Supplement: Supplementary file 1 — Supplemental information [file 41437_2024_676_MOESM1_ESM.pdf]

Supplementary material for:

Relatedness-based mate choice and female philopatry: inbreeding trends of wolf packs in a human-dominated landscape

Carolina Pacheco[1,2,3], Helena Rio-Maior[1,3], Mónia Nakamura[1,2,3], Francisco Álvares[1,3], Raquel Godinho[1,2,3]

[1] *CIBIO, Centro de Investigação em Biodiversidade e Recursos Genéticos, InBIO Laboratório Associado, Campus de Vairão, Universidade do Porto, Vairão, Portugal*

[2] *Department of Biology, Faculty of Sciences, University of Porto, Porto, Portugal*

[3] *BIOPOLIS Program in Genomics, Biodiversity and Land Planning, CIBIO, Campus de Vairão, Vairão, Portugal*

This PDF contains:

Supplementary Table S1 and S2

Supplementary Figure S1 to S8

SUPPLEMENTARY TABLE

Table S1. Set of 19 autosomal microsatellite loci used for individual identification of Alto Minho wolves, with the number of gene copies analysed, alleles per locus (AN), and observed (H_o) and expected (H_e) heterozygosity. Mean values (with standard deviation; s.d.) of all loci combined are also presented. *Loci with deviations from Hardy-Weinberg equilibrium ($p < 0.001$).

Locus	N gene copies	AN	H_o	H_e
AHT111	306	6	0.732	0.689*
AHT121	306	7	0.549	0.557*
AHT137	306	6	0.510	0.563
C04.140	305	4	0.724	0.665
C09.173	305	2	0.316	0.284
C20.253	306	5	0.810	0.733
C22.279	306	5	0.837	0.689
CPH9	287	5	0.451	0.404
CPH14	281	6	0.618	0.635*
FH2001	306	4	0.529	0.516
FH2054	302	4	0.447	0.439
FH2161	278	5	0.657	0.713
INRA21	306	6	0.758	0.706
INU030	302	6	0.440	0.447
INU055	287	6	0.791	0.758
Pez3	302	6	0.722	0.698
Pez5	302	5	0.738	0.678
REN169O18	301	3	0.436	0.410
REN247M23	240	5	0.395	0.487
**Overall mean**	297	5	0.603	0.583
**s.d.**	16	1	0.161	0.138

Table S2. Pearson correlation coefficients among eight genetic relatedness estimators and true values for relatedness using COANCESTRY 1.0 and ML-RELATE software. Analysis conducted on a dataset of 3000 simulated individual dyads representing six distinct relationships: parent-offspring, full-siblings, half-siblings, first cousins, second cousins, and unrelated individuals (500 dyads each).

	**TrioML**	**Wang**	**LynchLi**	**LynchRd**	**Ritland**	**QuellerGt**	**DyadML**	**MLRelate**	**TrueValue**
**TrioML**	1								
**Wang**	0.889	1							
**LynchLi**	0.884	0.987	1						
**LynchRd**	0.875	0.807	0.803	1					
**Ritland**	0.543	0.467	0.462	0.745	1				
**QuellerGt**	0.909	0.940	0.951	0.835	0.504	1			
**DyadML**	0.993	0.899	0.892	0.885	0.546	0.917	1		
**MLRelate**	0.629	0.570	0.564	0.571	0.374	0.575	0.628	1	
**TrueValue**	0.801	0.736	0.731	0.740	0.475	0.741	0.799	0.691	1

Likelihood relatedness estimators:

TrioML (Triadic likelihood estimator) described in: Wang, J. (2007). Triadic IBD coefficients and applications to estimating pairwise relatedness. *Genetics Research,* 89(3), 135–153. doi: 10.1017/S0016672307008798.

DyadML (dyadic likelihood estimator) and **MLRelate** are both implementations of the estimator described in: Milligan, B. G. (2003). Maximum-likelihood estimation of relatedness. *Genetics,* 163(3), 1153–1167. doi: 10.1093/genetics/163.3.1153.
The difference between the two estimators is that the former implementation allows for the presence of inbreeding while the former implementation assumes inbreeding to be absent.

Moment relatedness estimators:

Wang described in: Wang, J. (2002). An estimator for pairwise relatedness using molecular markers. *Genetics*, 160(3), 1203– 1215. doi: 10.1093/genetics/160.3.1203.

LynchLi described in: Lynch, M. (1988). Estimation of relatedness by DNA fingerprinting. *Molecular Biology and Evolution*. 5(5), 584-599. doi:10.1093/oxfordjournals.molbev.a040518.
Li, C. C., Weeks, D. E. and Chakravarti, A. (1993). Similarity of DNA fingerprints due to chance and relatedness. *Human Heredity*, 43(1), 45-52. doi:10.1159/000154113

LynchRd described in: Lynch, M., and Ritland, K. (1999). Estimation of pairwise relatedness with molecular markers. *Genetics,* 152(4), 1753-1766. doi: 10.1093/genetics/152.4.1753

Ritland, described in: Ritland, K. (1996). Estimators for pairwise relatedness and inbreeding coefficients. *Genetics Research*, 67(2), 175-186. doi: 10.1017/S0016672300033620.

QuellerGt described in: Queller, D. C., and Goodnight, K. F. (1989). Estimating relatedness using molecular markers. *Evolution* 43(2), 258-275. doi: 10.1111/j.1558-5646.1989.tb04226.x.

SUPPLEMENTARY FIGURES

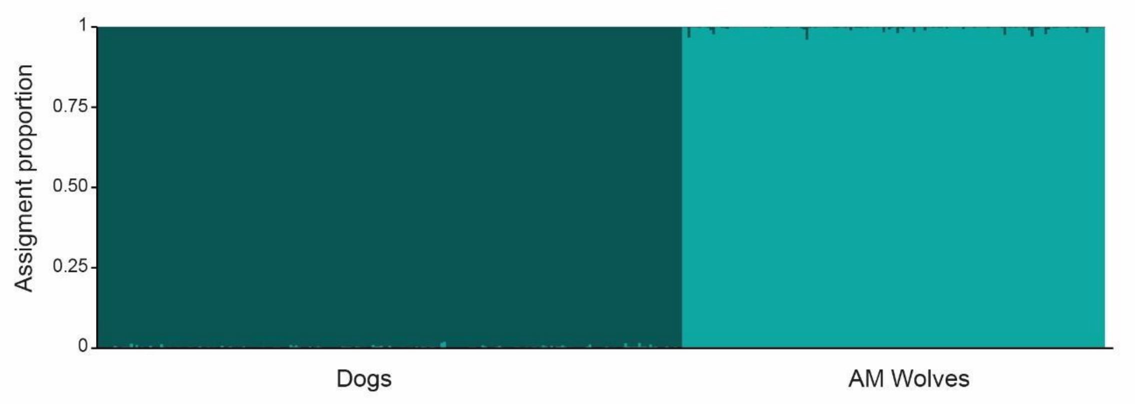

Figure S1. Individual assignment probability to two genetic clusters (K = 2) inferred by a Bayesian analysis using STRUCTURE software. Analysis was performed with reference dogs (213 individuals; Dogs) and wolves identified in Alto Minho (152 individuals; AM Wolves) for a set of 19 microsatellite loci. Each individual is represented by a vertical bar fragmented into two sections according to the individual's assignment proportion to the dog (dark shade) and wolf (light shade) genetic clusters.

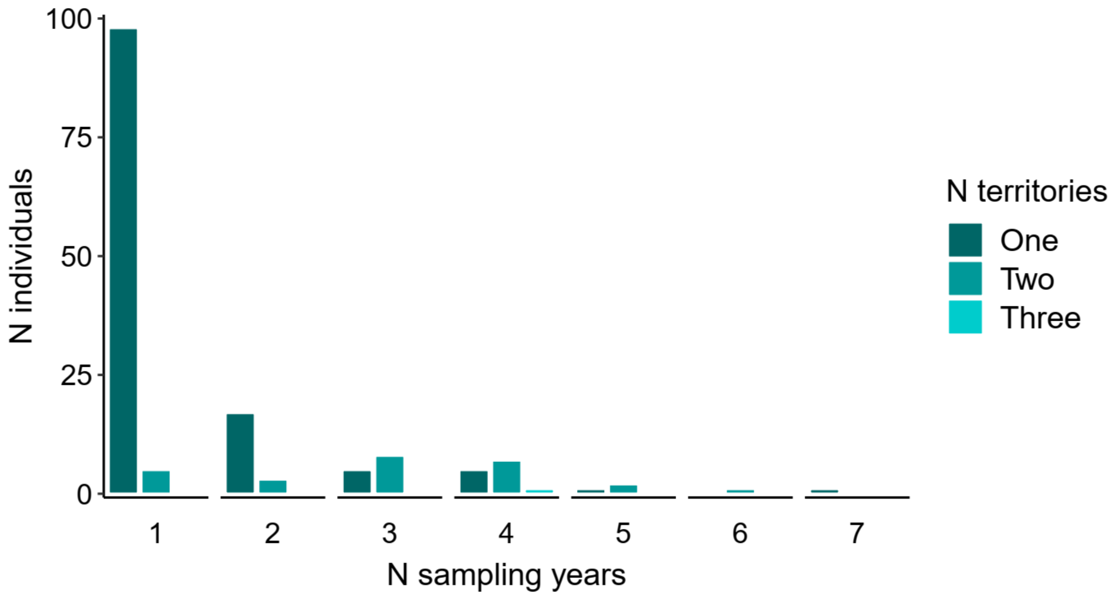

Figure S2. Number of sampling years and assignment to pack territories for each of the 152 Alto Minho wolves analysed.

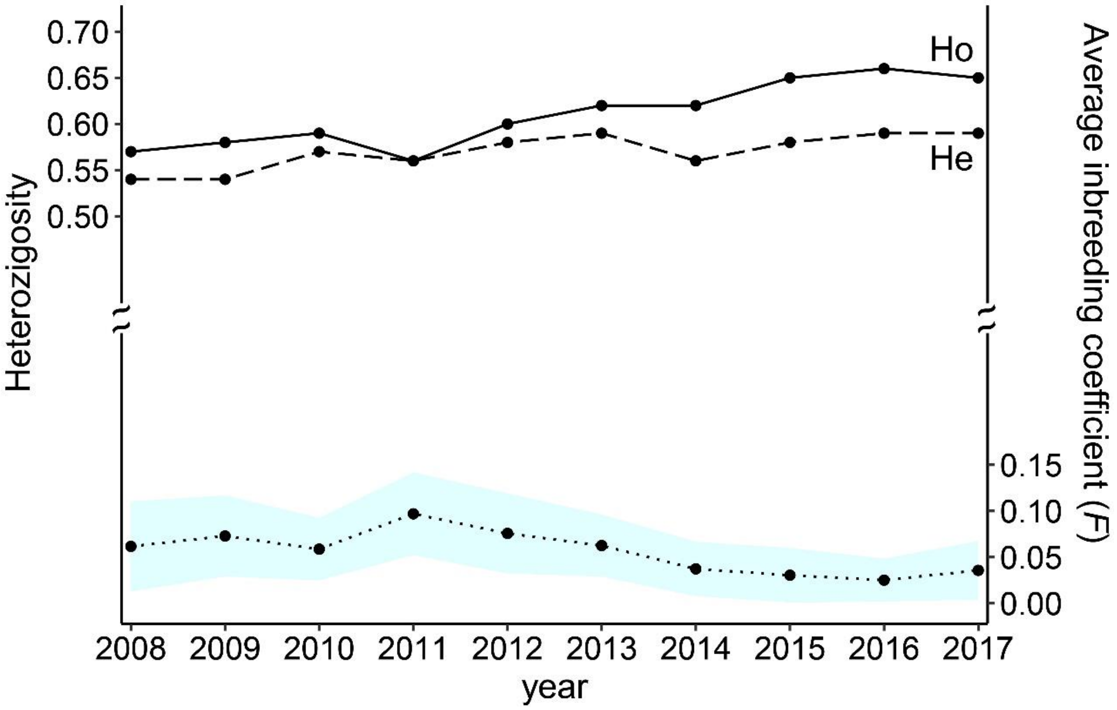

Figure S3. Temporal trend of observed heterozygosity (solid line), expected heterozygosity (dashed line) and average individual inbreeding coefficient (dotted line; ribbon indicate the standard deviation) based on all Alto Minho wolves detected in each sampling year (2008 - 2017). There were no significant differences between consecutive years.

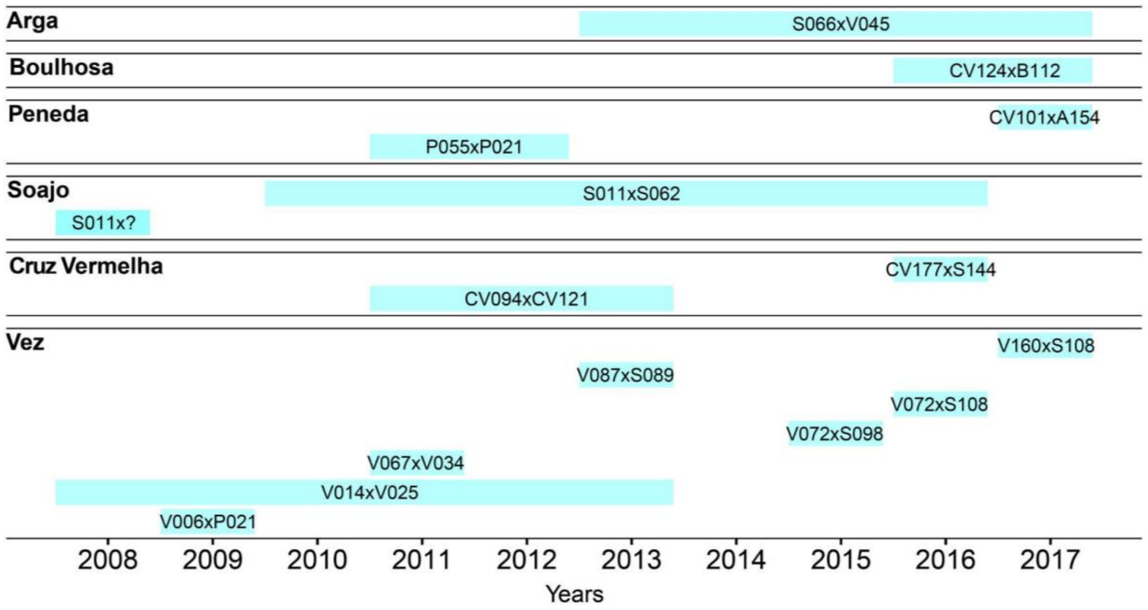

Figure S4. The time span of confirmed reproduction for each breeding pair per pack in Alto Minho. Only 15 breeding pairs are depicted, as it was not possible to ascertain the reproduction date of breeding pair P083 x CV094.

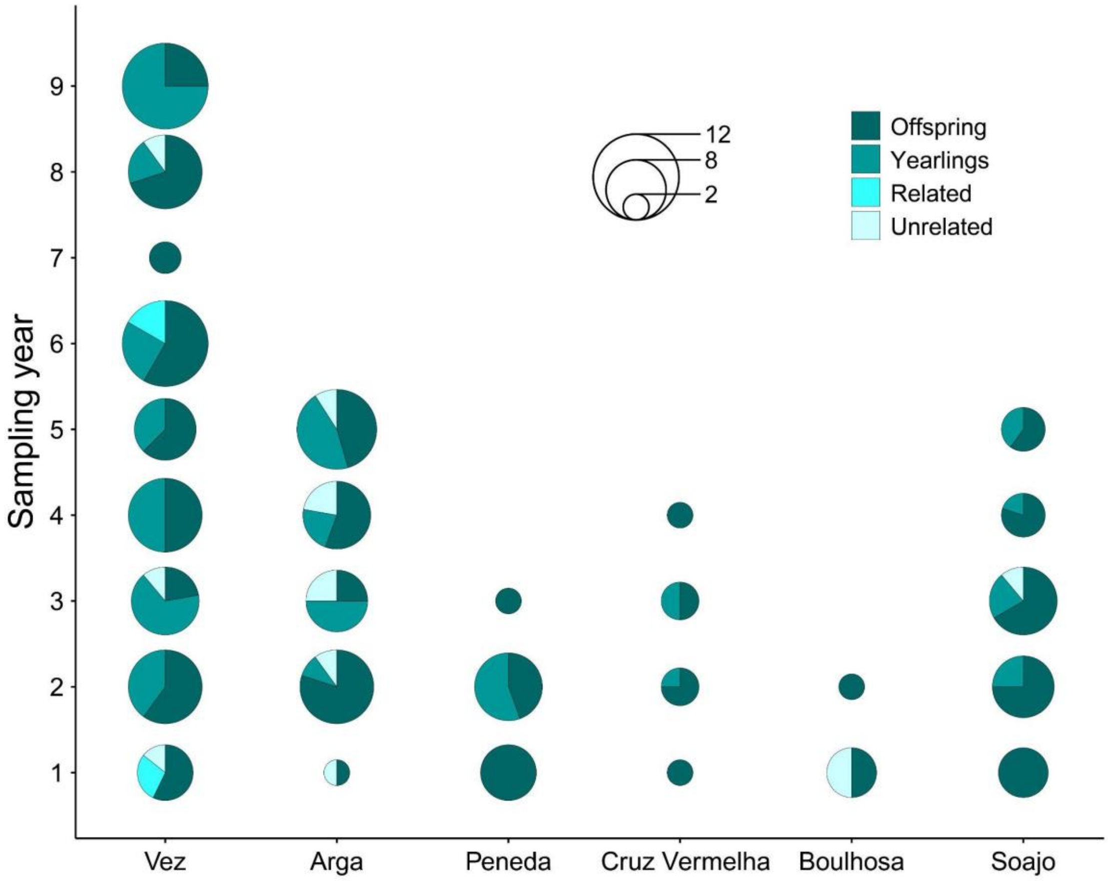

Figure S5. The proportion of offspring, yearlings, second or third relatives to the breeding pair and unrelated wolves detected in each pack/year with reproduction confirmed through genetic data, in Alto Minho.

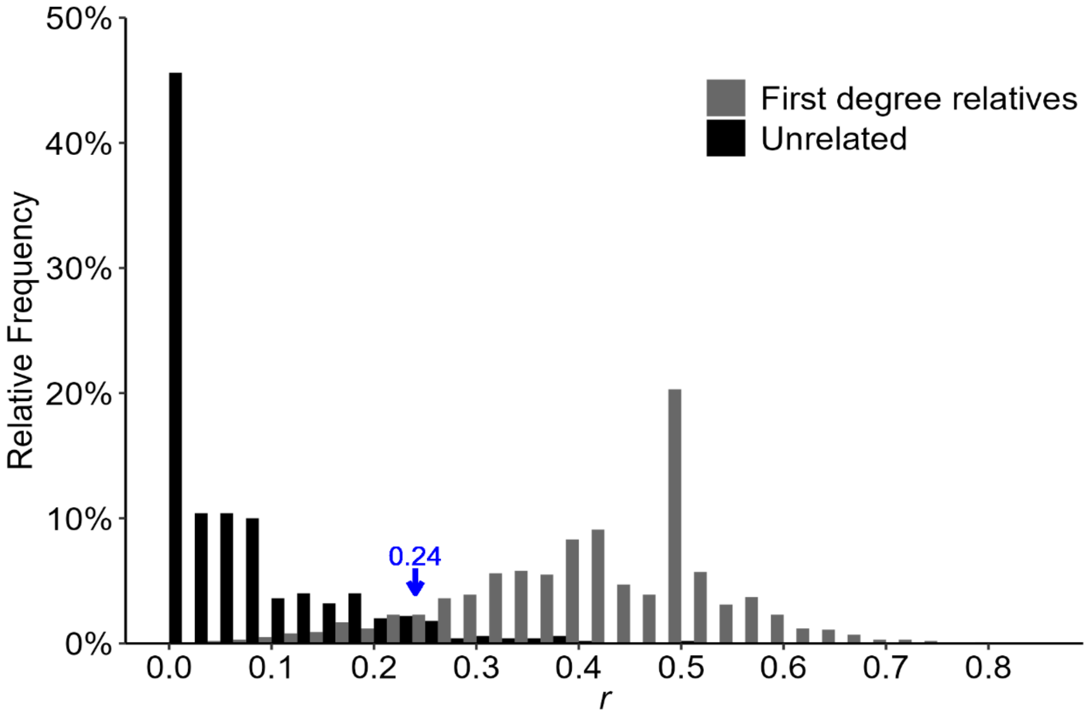

Figure S6. Distributions of relatedness (*r*) using the TrioML estimator for 1st-order relatives (parents–offspring and full siblings) and unrelated individuals obtained from 500 dyads simulated in COANCESTRY using allele frequencies of all Alto Minho wolves. The arrow indicates the midpoint value of relatedness (r = 0.24) between the averages of the two distributions used as a threshold for classifying a dyad as related. The expected proportion of misclassified parent−offspring or full sibling dyads as unrelated corresponds to 8%, whereas the expected proportion of misclassified unrelated dyads as parent−offspring or full siblings is 4%.

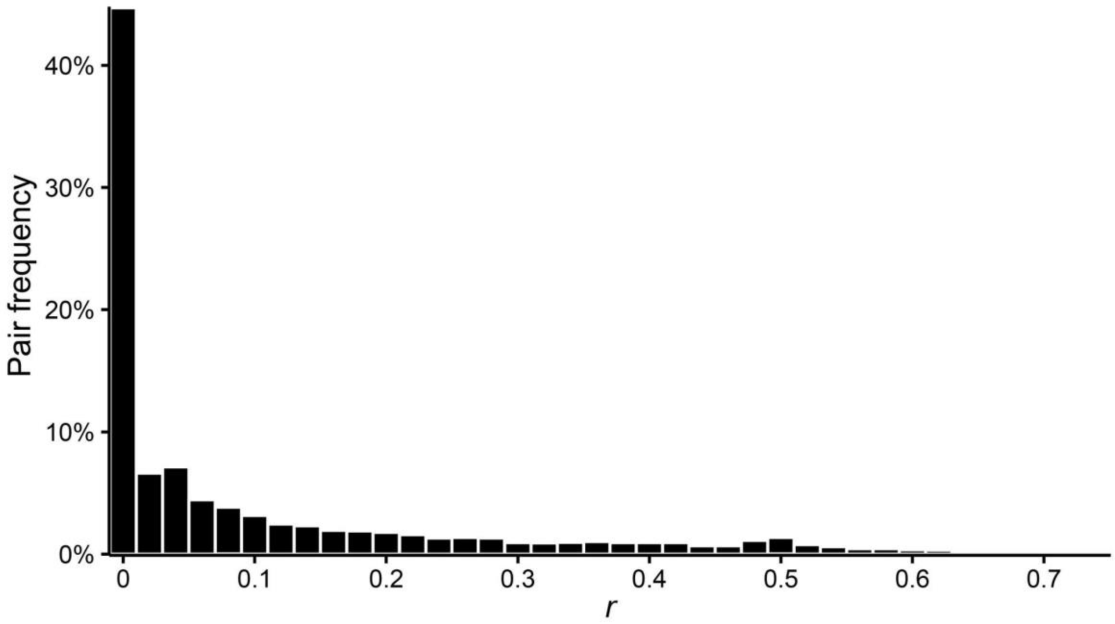

Figure S7. Distribution of the frequency of pairwise relatedness values for all wolves identified in Alto Minho. Values obtained with the software COANCESTRY.

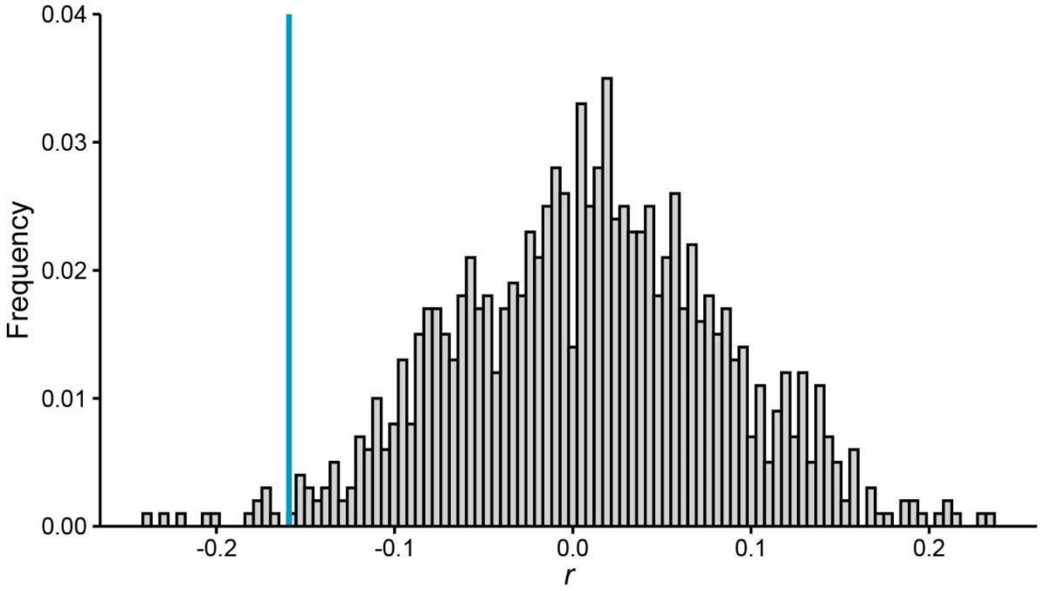

Figure S8. Distribution of the average relatedness values among the 1000 groups of 16 breeding pairs simulated with the software STORM. The blue line represents the average relatedness among the breeding pairs identified in Alto Minho.
